# Analyzing and predicting success of professional musicians

**DOI:** 10.1038/s41598-022-25430-9

**Published:** 2022-12-17

**Authors:** Inwon Kang, Michael Mandulak, Boleslaw K. Szymanski

**Affiliations:** 1 Rensselaer Polytechnic Institute, Network Science and Technology Center, Troy, NY 12180 USA; 2 Department of Computer Science, Rensselaer Polytechnic Institute, Troy, NY 12180 USA; 3 Społeczna Akademia Nauk, Łódź, Poland

**Keywords:** Computer science, Statistical physics, Complex networks

## Abstract

The emergence of streaming services, e.g., Spotify, has changed the way people listen to music and the way professional musicians achieve fame and success. Classical music has been the backbone of Western media for a long time, but Spotify has introduced the public to a much wider variety of music, also opening a new venue for professional musicians to gain exposure. In this paper, we use open-source data from Spotify and Musicbrainz databases to construct collaboration-based and genre-based networks. We call genres defined in these databases primary genres. Our goal is to find the correlation between various features of each professional musician, the current stage of their career, and the level of their success in the music field. We build regression models using XGBoost to first analyze correlation between features provided by Spotify. We then analyze the correlation between the digital music world of Spotify and the more traditional world of Billboard charts. We find that within certain bounds, machine learning techniques such as decision tree classifiers and Q-based models perform quite well on predicting success of professional musicians from the data on their early careers. We also find features that are highly predictive of their success. The most prominent among them are the musicians’ collaboration counts and the span of their career. Our findings also show that classical musicians are still very centrally placed in the general, genre-agnostic network of musicians. Using these models and success metrics, aspiring professional musicians can check if their chances for career success could be improved by increasing their specific success measures in both Spotify and Billboard charts.

## Introduction

At every age and genre of music, there is a small fraction of professional musicians who achieve success. Although the definition of success often varies, many research efforts have been focused on discovering measures that can be used as proxies for success and on understanding the reasons behind the validity of these measures. Past works have taken this approach in various subject domains, such as art^1^, movies^2^ and in research^3^ and have presented models for predicting the success of a given individual.

Considering the music field specifically, past works utilize Spotify’s database to analyze the popularity of individual songs^4^ or to construct networks of professional musicians and analyze the relationship between popularity and the network rank^5^. The latter use these network models to capture the interactions of nodes, most often from a collaborative or relational perspective.

This work aims to contribute to this line of research on analysis and predictions of the success of professional musicians by using together the Spotify, MusicBrainz and Billboard’s datasets. We analyze the careers of professional musicians using several features, such as music genres, productivity (quantified as the number of releases), release trends, and collaborations with other musicians. We show that the success of a professional musician defined by the follower count and popularity score can be determined with some precision by a complex relationship between multiple features defined in a collaboration network. We also find that traditional success measures such as appearing in Billboard’s Hot 100 list can be predicted accurately with a simple classifier using the collaboration features that also provide guidance for aspiring professional musicians on how to advance their networking profile. Using this knowledge, we then try to find what features and to what extent a musician should improve to become more successful in both digital and analog domains.

These predictions are further refined by exploring a dependence of musician popularity on genres of music released by this musician revealed under a modified *Q*-model^3^, showing a complex relationship between success, genre, and several logistic properties of top artists. In both cases, we apply a variety of classifier models and show that the weighted accuracy rises up to 77% with tree-based classifiers using a combination of these properties, demonstrating these models’ ability to predict success among top musicians under Spotify-generated data. We then show that the task of predicting whether a musician will be featured in Billboard’s Hot 100 list only by looking at the features of their collaborators can be learned with a tree-based classifier with weighted accuracy reaching up to 77%. We also find that collaboration with a high-profile musician is not always necessary for a musician to achieve success of appearing on a Billboard’s Hot 100 list.

The main contribution of this work is developing methodologies for:

1. Creating network profiles of professional musicians based on their features and finding the features that most strongly correlate with success.
2. Selecting a machine learning model convenient for designing *classifiers* for interpretable predictions of the success of a musician.
3. Clustering all musicians into a predefined number of clusters C, each cluster with a centroid profile defined by averaging features of the cluster members, and then randomly perturbing features to create many additional synthetic profiles.
4. Finding profiles that improve the most chances of musicians in the cluster to achieve success in the future, since *the found profiles predict the ways a musician can achieve success by improving its features.*

## Related works

As a preface to our work exploring trends of success among modern professional musicians, we review similar efforts in quantifying the features to a variety of disciplines. Within the field of science of science, Wang et al. propose a temporal model for citation patterns within research publications, showing similar release trends among successful papers^6^. Sinatra et al. extend this into a success-citation model, showing promising results towards quantifying and predicting scientific impact^3^. Success can also be studied in a focused manner, such as through trends on Nobel Prize recipients^7^. Apart from success, similar properties are evaluated using publication data, such as regularity within research interest growth over a career^8^ and the quantification of such growth relative to success in a competitive setting^9^.

Outside of the science of science field, similar methods and efforts are applied towards the quantification of success through specific properties in the fields of art^1,10^, show business^2,11^ one-hit wonders in creative fields^12^ and within the music industry, both for success distribution and collaboration^13,14^. Another work^15^ also uses mentions in social media to predict the success of a newly released album. Within all these efforts, generalized models are developed and trends are noted, commonly specific to the field and the properties studied, allowing for some basic prediction. Our work aims to contribute to these efforts by studying modern music within Spotify and applying prediction methods, also studied by South et al.^5^. Unlike the former, our work focuses on top Spotify musicians and the application of classification models for prediction using a genre dataset in which each musician is assigned genres of their music and a collaboration network of musicians.

## Preliminaries

### Data

We first detail our collection of data. Our work is based on data from two datasets derived from a combination of data queried from the MusicBrainz database^16^ and data collected using Spotify’s API through the Spotipy Python package^17^. MusicBrainz is an open-source music database that contains release information on about 1.9 million professional musicians and around 3 million releases as of August 2021. Using these two databases, we were able to gather data on musicians and music created between 1890 and 2020, including Spotify’s assignment of musicians to a subset of 3031 genres of their music. We refer to this data as a genre dataset. To focus on the release patterns within a specific market, we restrict the region of releases to the United States of America for both the collaboration network and the genre dataset. Our final dataset contains information scraped from these two sources about professional musicians and their collaborations in their releases. In Musicbrainz, an *artist* is defined as either a performer who partakes in playing and recording music or a composer of the performed music. A *release* refers to each unique commercial release of a musical product by an artist or a group of artists. It can be a single or an album, and each release is counted separately on different mediums, such as digital or physical copies. Since the data scraped from Musicbrainz is associated with the Spotify data, our dataset only contains the digital releases, especially the releases made on Spotify. Here, for clarity of what art we refer to, we replaced a generic term *artist* with a more specific *professional musician* sometimes abbreviated to *musician*.

After the data collection process is completed, the final dataset is ordered by the time the musicians were added to the network. This order does not reflect the actual time sequence of information about the musicians. Hence, we sort the final dataset in an ascending order of the ‘last release’ feature that orders the musicians by the time they received their most recent popularity score included in the dataset. Once the dataset is sorted, we split the dataset into training, validation, and testing data subsets. With this order, we can use the popularity scores in training and validation subsets to learn the pattern of successful musicians and test it on the testing data. Hence, the training and validation data subsets are used to train each classifier.

The final dataset contains musicians with the dates of their last releases ranging from 1969-01-01 to 2020-12-25. We put first 60% of the musicians with the earliest Spotify popularity scores in the training subset, and the last 30% of musicians with the latest Spotify popularity scores in the testing subset. The remaining musicians in between these sets are placed in the validation subset. Consequently, the range of the last release date of musicians in the training subset extends from 1969-01-01 to 2018-12-31, with 7743 musicians. The range in the validation subset starts from 2019-01-01 and ends in 2019-10-17 with 1290 musicians, while in the testing subset, this range covers the period from 2019-10-18 to 2020-12-31 with 3872 musicians. As a result, the training, validation, and testing dataset do not overlap and contain 7,743, 1,290, and 3,872 musicians, respectively.

### Musicians collaboration network

As South et al.^5^, we use a dataset to build a collaboration network of professional musicians. This dataset is initially seeded with a list of the top 1,000 musicians by their stream count, provided by https://Chartmasters.org. Pulling data from Musicbrainz, we associate these musicians with other musicians based on their releases to develop a collaboration network. Data collection continued until the network had no more new edges, yielding 114,955 musicians and 27,359,052 releases. Artists that did not have a Spotify profile were filtered out from the data. The resulting dataset contains 22,517 musicians and 595,849 unique releases, disregarding duplicate releases outside of the US. For each release, we gather the release date, associated label and region from MusicBrainz and collect the release musicians’ popularity scores and numbers of followers from the Spotify API.

**Figure 1:**
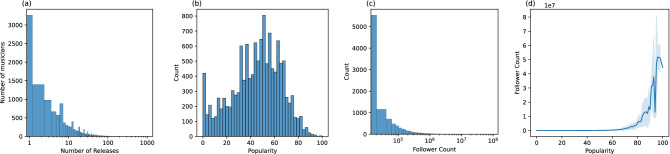
(**a**) Release Count distribution of musicians. (**b**) Popularity score distribution of musicians. (**c**) Follower Count distribution of musicians. (**d**) Follower count associated with popularity.

The counts of numbers of releases for musicians in the collaboration network are mostly low. As seen in Fig. 1a, 90% of the musicians in the dataset have no direct releases. This is to be expected since our data collection method focused on the collaboration history of individual musicians regardless of their release counts. About 40% of musicians have at least one and at most three official releases. This means that in our dataset only $6\%$ of musicians have four or more releases. The distribution of popularity and follower count can be seen in Fig. 1b and c. Figure 1d shows the correlation between the popularity score and follower count. As expected, musicians with a higher number of followers tend to have a higher popularity score. But these scores are not strictly linearly correlated as they point to slightly different measures.

#### Popularity score

The popularity score is a metric provided by the Spotify API, with values ranging from 0 to 100. It is a measure used and calculated internally to rank professional musicians by their perceived popularity at a point in time. This score is affected by the number of plays a musician has, as well as how recent those plays were. This metric provides an insight into the musicians’ success at the time of measurement. Unfortunately, the historical data of musicians’ popularity scores are not publicly available. Thus, we cannot track the change of popularity of the musicians. Despite this, we use this score as a metric for viewing how popular a musician currently is due to Spotify’s usage of the measure as a ranking baseline.

#### The number of followers

Spotify also provides the number of followers a professional musician has. This value can be any non-negative number, ranging from 0 to several million. These numbers are defined by an internal function of the Spotify platform that allows users to create follow links to musicians that trigger Spotify notifications to such users about new releases by so followed musicians. This measure is a relative indicator of how ‘popular’ a musician is and provides a baseline comparison metric between multiple musicians. Furthermore, since this value is not affected by time unlike the popularity score which factors in *recent* streaming counts, we can view the follower count as a more temporally robust measure of popularity compared to the Spotify popularity value. While this value is subject to change as users follow and unfollow musicians, such updates cause minor changes to overall rankings among top musicians.

#### Billboard’s Hot 100

While Spotify’s API provides users with two metrics to gauge professional musician’s success, high values of these metrics do not necessarily translate into traditional success. Each week, the Billboard, an American music magazine, publishes a list called Billboard’s Hot 100, which ranks the one hundred most successful songs in terms of two features, albums sales and radio plays. Spotify does not consider these two features when calculating their metrics. Since Spotify currently dominates in digital streaming of music, it can be argued that the features from Spotify are sufficient measures of a professional musician’s success. However, digital streaming suffers from a wide variety of manipulations from its users, such as fans of a particular musician boosting their idol’s stream counts to place higher in Spotify’s rankings. For this reason, we also use the Billboard’s Hot 100 ranking and its correlation to Spotify popularity and other features of a musician to find more reliable measures of success in the music business. To associate the musicians in our dataset with the Billboard’s Hot 100 rankings, we use the weekly ranking data between the start of the dataset, August 1958, and November 2020 that includes 3256 Billboards. Another paper focusing on predicting success in the music fields^15^ uses the Billboard’s Hot 200 list as the target metric. This list contains the ranking of albums that were the most popular based on sales metrics. This list is well suited for predicting the success of newly released albums because it ranks the entire album rather than a single song. However, we have opted to use Billboard’s Hot 100 list because it selects only the top 100 rather than 200, thereby making it a more ’prestigious’ metric for a professional musician better reflecting their current popularity.

**Figure 2:**
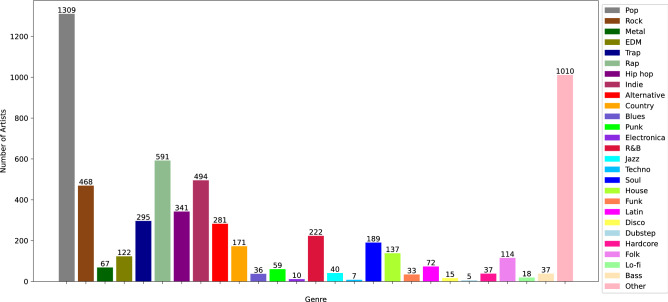
Distribution of the genre dataset (with total of 2363 musicians).

### Release genre

To separately consider the impact of genre on popularity of professional musicians, we extract data based on shared genres using methods applied to the first dataset to generate a secondary dataset. Pulling data from Spotify’s listing of the daily top 200 songs from January of 2017 to November of 2021, we collected 2,363 professional musicians with primary releases in the United States of America. We then list basic genres in the decreasing order of the number of musicians who released music in each primary genre. We select such several genres *g* that the “other” genre, which contains all genres with the rank equal or higher than *g*, has fewer musicians releasing music in these genres than the most popular genre has. In our dataset, $g=27$. We refer to $g-1$ primary genres from the list and the “other” genre, as normalized genres. Then, we assign to each musician *m* a binary vector $G_m$ of length *g* with value one at position *p* if the musician has released music in the *p* normalized genre and zero otherwise. The distribution of the numbers of musicians over the normalized genres is shown in Fig. 2.

## Background

Machine Learning is commonly used for classifying dataset entries with attributes which are used as features for classification. Here, we apply it to the musician’s datasets collected by the music industry. In this section, we outline the methods used in our work to analyze the success of professional musicians. The main machine learning model used in this work was a single XGBoost^18^ tree. Our initial experiments also used Logistic Regression^19^, Random Forest Classifier^20^ and Support Vector Machines^21^. These models were chosen for their interpretable nature, which allows us to understand the inner workings of each of these models and present its results in an easily digestible manner. However, we found that only XGBoost was able to maintain interpretable character and at the same time to yield meaningful results with a weighted accuracy (a variant of classic accuracy score adapted to imbalance data sets we use for predicting success of musicians) greater than 50%. Consequently, the rest of the analyses in our paper use only this model. We used Python’s xgboost package^18^ to run XGBoost and Python’s scikit-learn package^22^ to run the rest of the models.

Our models are binary classifiers predicting which musicians in a dataset will achieve success and will belong to a positive class of size P, and which will remain aspiring to success and stay in a negative class of size N. In our case, the classes are imbalanced since P is from 5 to 20 times smaller than N ^23^. Therefore, we cannot use the classic *accuracy metric* to measure the performance of our models. Instead, we are using the *weighted accuracy* (WA) which is an average of sensitivity and specificity. Sensitivity, also known as recall, is the fraction of positive instances that were correctly predicted among all predictions for P positive cases. Specificity is a fraction of correctly predicted negative instances among all N negative cases. Thus, weighted accuracy balances quality of predictions in both imbalanced subsets of positive and negative cases. Another balanced measure of performance is the F1 score computed as a harmonic mean of the recall and precision. The latter computes the fraction of correct positive predictions among all positive predictions. These metrics can be expressed succinctly with additional variables TP, FP, TN, FN, denoting true positive, false positive, true negative and false negative predictions, respectively.

$$\begin{aligned}{} & {} Sensitivity, recall = \frac{TP}{P}; \quad{Specificity = \frac{TN}{N};} \quad{WA = \frac{TP}{2P}+\frac{TN}{2N};}\quad {Precision = \frac{TP}{TP+FP};} \\ &\quad F1 = \frac{2}{1/Recall + 1/Precision}. \end{aligned}$$

To learn the best performing parameters, each classifier tree uses the training subset of each dataset as a basis for predictions of ground truth data in a validation subset. The qualities of the resulting models are evaluated by comparing predictions made using each validation subset to the corresponding testing subsets ground truth data.

For another set of experiments, we create synthetic profiles of professional musicians by using K-means algorithm to generate C = 300 clusters from feature vectors of all professional musicians. Our aim is to provide guidance to musicians for improving their careers. Thus, we find the minimal set of changes in features of a musician that are needed to make this musician successful.

The final anonymized datasets of musicians and their features are available in a public github repository (https://github.com/inwonakng/predicting-musician-success), along with the codes for training and evaluating the models and running the synthetic profile experiments.

## Methods

### Professional musicians analysis

#### Collaboration network

The music industry is heavily dependent on collaboration. An unknown professional musician can achieve fame quickly by collaborating with a well-known musician. The form of collaboration may not always be mutual, as in some cases, a musician can re-interpret or be inspired by music created decades, or even centuries before. The information about collaborations between musicians can contain many nuanced details about their success. Hence, we construct a network of musicians linked by collaboration and extract features of such networks to be used in predictions.

**Figure 3:**
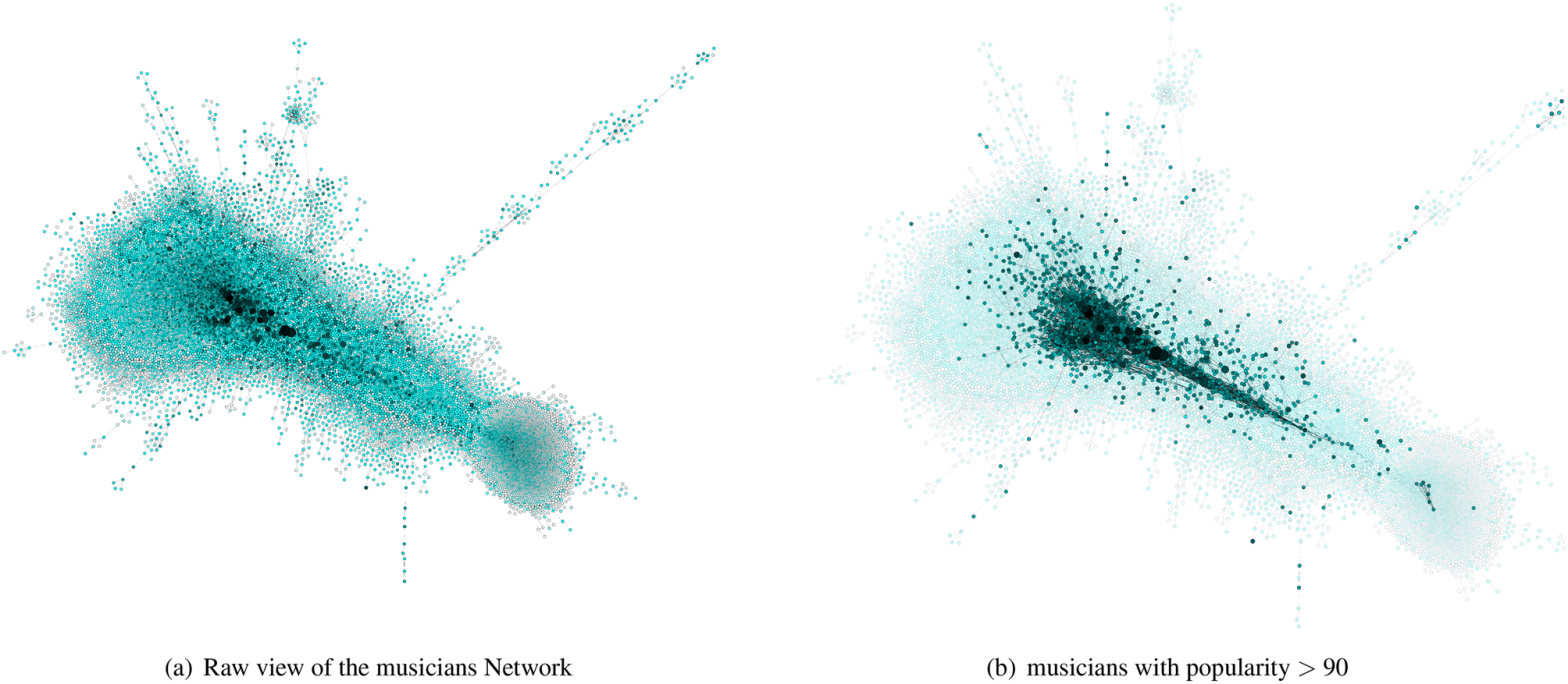
Musician network views for collaboration analysis.

More formally, a collaboration network $G_M$ between professional musicians is a directed weighted graph whose nodes represent a set of professional musicians, *M*, while each edge in set *E* is drawn between a pair of musicians $M_A,M_B$ when musician $M_A$ has released records featuring music of musician $M_B$, while $w(M_A,M_B)$ denotes the edge weight that represent the number of times $M_B$ has been credited in a release made by $M_A$. Our professional musicians network is composed of 22,509 nodes and 595,849 edges. For the success metrics of each musician, the follower count ranges from 0 up to 90 million while the popularity score is between 0 and 100. An overview of this network can be seen in Fig. 3a and  b.

#### Network rank

To assess the correlation between musician’s position in the network and their various measures of success, we use a network rank measure based on eigenvector centrality. Since the popularity score ranges between (0, 100), we simply group the musicians of equal popularity scores together and measure the standard deviation and mean of their network rank scores. Although the popularity score is convenient, its drawback is being a single value. In contrast, the follower count of each musician is a sequence of values measured at the same time for all musicians. To consider the network rank score at various follower counts, we create 100 synthetic bins to group the musicians together, since the values do not have a set bound. The follower count has an exponential distribution, with most musicians on the lower end and only a few at the top. This follows the trend noted by J.A. Davies^13^ in that the distribution of success among musicians tends to be spread exponentially. To normalize this data, we apply a logarithmic function to the number of followers and then categorize them at 100 intervals evenly divided between the minimum and maximum value. In both comparisons for popularity and follower count with the musicians’ network rank, we observe that while some musicians in the upper-mid range musicians tend to have the highest network rank, it does not have a strong correlation, as shown in Figs. S1 and S4 in Supplementary Materials.

Interestingly, the musicians who have the highest network rank scores are classical composers. The top three composers are Wolfgang Amadeus Mozart, Ludwig van Beethoven and Johannes Brahms, names that are familiar even to those who are not avid classical music listeners. After filtering out classic composers by using the genre tags, we found that most musicians with high network rank scores were instrumental musicians, such as the London Symphony Orchestra or Philharmonic Orchestra. In fact, when examining the genre tag mentioned at the top 100 network ranked musicians, we find that every genre is either classical or instrumental. The fact that musicians who produced/composed music over 200 years ago are still dominating the collaboration network can appear strange at first. Yet, this is understandable considering that sampling, which is an act of borrowing a piece of music from someone else while giving them credit, is now a prevalent method of digitally producing music. At the time of writing, neither Spotify nor Musicbrainz offered information on the type of collaboration, so we were not able to construct a more detailed network with different types of edges.

#### Musician classification using Spotify

After observing that there indeed is a pattern among popular/widely followed musicians and their releases, we build a prediction model to identify musicians who can be considered successful.

To each musician *M*, we assign 10 features derived from the Popularity Score, and the Number of Followers, as well as the same 10 and another 9 features derived from the Billboard appearances. All the assigned features are listed in Table S1 in Supplementary Materials.

For predicting the popularity score and follower count, we leave out the features related to other Spotify metrics to remove bias in our classifiers. A preliminary check of our features calculated Kendall Tau’s distance between each pair of features to measure the normalized correlations between this pair (Fig. S4 in the Supplementary Materials contains the computed Kendall Tau’s coefficients matrix). The test shows that the two success measures—popularity and follower count—are highly correlated. Hence, we remove one of these metrics from the feature set when building a classifier for the other. Features used for each target variable are marked with a $\checkmark$. Given these features, we set to build three classifiers that can predict the popularity score ($M_{pop}$), follower count ($M_{fol}$) and the number of Billboard appearances. South et al.^5^ use a model called Social Group Centrality, which groups musicians into different bins, such as celebrity, community leaders and masses based on their popularity and network rank. In our case, we are more interested in finding a model to predict the success metrics given the node features. Thus, we consider some well-known shallow machine learning models to learn the problem of predicting success. We only chose to look at shallow models because of their transparency, so that we can further understand model learning patterns and the relationships between features.

We also limit the features that are used for each classifier’s training. For the popularity score and follower count tasks, we leave out the features correlated to the collaborators’ popularity and follower count. In our experiments, we find that the classifiers are trained with a higher accuracy when these features are included. However, the resulting tree only considers these collaborator features, and does not provide any useful information other than that ‘successful musicians collaborate with each other’. In this work we are focusing on finding any other patterns in successful musicians, so we do not use the correlated features in the training process. These features are only included in the Billboard’s Hot 100 list prediction, as this task’s domain is outside of the scope of Spotify and providing this task with more features increases the quality of its predictions.

We first build two classifiers to predict the popularity and follower count of each musician. Then build a classifier to predict whether the musician will appear in a Billboard’s Hot 100 list.

### Defining success

In our dataset, we have three different metrics for success of each professional musician – their popularity score, the number of followers (both on Spotify), and the appearance on the Billboard’s Hot 100 list. Usually, success is defined as being outstanding in the field, and in this paper, we are interested in whether the musician is successful or not. For appearing on the Billboard metric, no filtering is needed, since only 100 musicians can appear on the list at any given week.

We need to establish grand truth values for musicians in all our datasets and subsets. The most objective and clearly defined seems to be an appearance on the Billboard’s Hot 100 list. The list is selected weekly by a team of professionals using consistent ranking. For all three datasets defining major musician’s metrics, Billboard’s Hot 100 appearances, popularity score, and follower count, we proceed in two stages. In the first stage, training, we use only training data features and sets of parameter values in search of parameters that yield the best performance. To assess this performance, we establish the ground truth in validation data, first in Billboard’s Hot 100 dataset, by labeling each musician with 1 if this musician has ever appeared on Billboard’s Hot 100, and 0 otherwise. We also set a threshold, $\sigma _v$, to measure the fraction of musicians who achieve success among all musicians in the validation set. This enables us to map Billboard appearance distinction to other metrics. Thus, for the other two datasets, we label a musician with 1, when the musician’s rank under the considered metric corresponds to the $\sigma _v$ fraction of all musicians, and zero otherwise. Finally, we evaluate the prediction performance by measuring how close the predictions are to the validation data ground truth.

In the second stage, testing, we first produce the predictions using features of musicians from validation subset. Then, we label musicians in testing set as above, using appearance on the Billboard’s Hot 100 for one dataset, and thresholds in other two datasets, as described above. This is done after the predictions were made, so we are allowed to access the testing data subset.

We counted the fractions of musicians who achieved appearance in each Billboard data subset. We found that these fractions are $f_{te}=19.37\%, f_{v}=13.88\%,$ and $f_{tr}=11.22\%$ in training, validation, and testing subsets, respectively.

Following these metrics, we then establish such fractions for popularity score and follower count databases. Using the same method as above, we found that the positive labels for popularity score make up 36.96%, 20.54%, and 11.73% of each training, validation, and testing subsets, respectively, while for the follower count, the corresponding percentages are 31.02%, 18.45%, and 11.22%.

### Predicting success measures

#### Predicting popularity score

We use the established ground truth thresholds as follows. For training, we use $\sigma _v$ to measure the performance of the candidate tree and to select the best parameters. For testing, we also use $\sigma _v$ as the best estimate of the ground truth for testing data, while true value of it $\sigma _{tr}$ is used for scoring the performance of the prediction of the correct label for each musician. To evaluate different types of shallow machine learning models, we considered logistic regression, SVM, Random Forest and XGBoost classifiers. We chose these models because they are either simple or tree-based structures. This allows us to better understand each model’s ability to identify the success of musicians.

In practice, we found that using linear models does not work with our data. This agrees with our observations that there are no linear correlations between the features. The ensemble models (Random Forest) also were not able to learn from the data, producing high training accuracy but falling below 50% in the testing step, suggesting high overfitting. However, we found that a single XGBoost tree is well-suited to our data. Even with heavy imbalance between successful musicians and those who are not, the XGBoost classifier was able to classify the musicians with a decent weighted accuracy score 70% and F1 score of 63% in testing., while the other models classified performed worse than 50%.

To compensate for the fact that the number of musicians who had a popularity score above this threshold was very small compared to those who did not, we boosted the weight of the positive samples in the training data. For the number of positives, *P* and number of negatives *N*, the weight is calculated as *N*/*P*.

We also conducted hyperparameter tuning for the XGBoost classifiers to find the best setup for each success metric. We focused on limiting the tree size—as our main goal was to produce an interpretable decision tree—and on correctly identifying the key features used in the decision process. Accordingly, we aimed at getting XGBoost’s internal parameters $\alpha$ and $\lambda$ that keep the complexity of the tree low by making the model more conservative and limiting the *max_depth* parameter to control the overall depth of the tree. Tables S2 and S3 in Supplementary Materials contain the range and final values of optimal hyperparameters found for each metric. To ensure the experiment’s reproducibility, the random state of each tree was 0 at every training step. Because XGBoost outperformed every other classifier we considered, we looked more into using this classifier to examine the features of a popular musician. XGBoost can be used like an ensemble model, training *N* trees and aggregating their predictions. We found that reducing the number of trees in the classifier improves the performance, suggesting that there is an underlying simple structure that is being estimated by the many trees.

**Figure 4:**
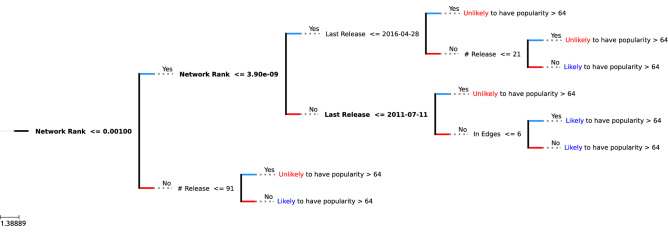
A Visualization of XGBoost tree classifying popularity > 64.

A visualization of the tree is shown in Fig. 4, with the top three features marked in bold. Note that the feature values may not reflect the real-life values, as they were normalized ahead of time. We also constrained the max depth of the tree to create a model that can be easily viewed and understood. We found that restricting the tree to the depth of at most three yielded the best result.

Although we use the XGBoost tree as a binary classifier, the prediction value at each leaf node is expressed through a continuous range from the positive (successful) to the negative (unsuccessful) labels. Hence, all positive labels are encoded as 1, while the negative ones are encoded as -1. Thus, each leaf node of the tree ends up with a value of either 1 or -1.

According to the results, we can make the following observations:

- If a musician has network rank **lower than** $\mathbf {3.9 * 10^{-9}}$, has released music **after 2016**, and has **more than 21** releases, they are likely to have popularity score **above** 64.
- If a musician has network rank **lower than 0.001** but **higher than** $\mathbf {3.9 * 10^{-9}}$ and has released music **after 2011**, they are likely to have popularity score **above** 64.
- If a musician has network rank **higher than 0.001** and has **more than 91** releases, they are likely to have popularity score **above** 64.

It is interesting to note that network rank seems to be the most relevant feature for musicians to have higher popularity. When the network rank is low, the tree also favors *younger* musicians with *more* releases, as can be seen in the first observation.

#### Predicting follower count

**Figure 5:**
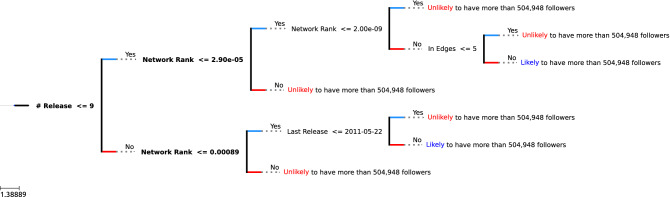
A Visualization of XGBoost tree classifying follower count > 504,948.

Following the same procedure as training for the popularity score, we get $\sigma _{f\_tr} = 459,442$ and $\sigma _{f\_v} = 504,948$ Our classifier showed steady performance throughout the cross-validation step, resulting in the final weighted accuracy of 71% and F1 score of 60% in testing.

Analyzing the nodes in Fig. 5, we make these following observation:

- If a musician has **less than 9** releases, has network rank **lower than** $\mathbf {2.9 * 10^{-5}}$ but **higher than** $\mathbf {2 * 10^{-9}}$, and has **more than 5** collaborators, they are **likely** to have more than 504,948 followers.
- If a musician has **more than 9** releases, has network rank **lower than 0.00089**, and has released music after **2011**, they are **likely** to have more than 504,948 followers.

Unlike the popularity tree, we observe that the first node considered is the number of releases. Then the second most important feature appears to be the network rank. As can be seen in observation 2, if the musician has a lower number of releases, then the network rank must be high for them to meet the success criteria.

#### Predicting appearance on Billboard’s Hot 100

**Figure 6:**
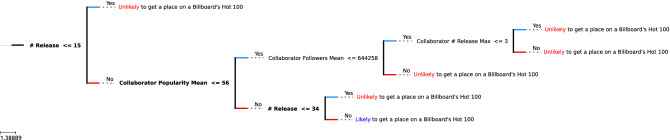
A visualization of XGBoost tree predicting musician’s appearance on a Billboard’s Hot 100 list.

After building a classifier for each of Spotify’s provided metrics, we build a final classifier on predicting whether a musician will appear on a Billboard’s Hot 100 list using the same collaboration network. We used the Billboard’s Hot 100 list because it is widely accepted as a benchmark of success in a musician’s career. While Spotify’s metrics provide an insight of trends within the streaming business, we are interested in finding out if we can translate this to a more traditional metric of success which is the Billboard’s rankings. Our dataset, including the popularity score and follower count, contains data on musicians up to 2020-12-31. So we only consider the Billboard lists up to this date. We used the dataset provided by Kaggle^24^ that contains songs that appeared in at least one of the Billboard’s Hot 100 lists from 1958 until November of 2021 and extracted the Billboard rankings until 2020-12-31.

To focus on the collaboration features of the musicians rather than their inherent features, we excluded the musicians’ own popularity and follower count in the considered feature set. Instead, we added extra features that described the collaboration history of the musician, such as the standard deviation, average and maximum of their collaborators’ popularity and follower count. Using the same settings as previous classifiers, we were able to learn this task with a weighted test accuracy of 79%, outperforms our previous two trees.

The learned decision tree can be seen in Fig. 6 with the top three features marked in bold.

It is interesting to note that we can predict the musician’s appearance on a Billboard’s Hot 100 list without looking directly at their Spotify popularity and follower count. Looking at the resulting classifier shown in Fig. 6, we draw the following conclusions:

- If a musician has **more than 15** releases, has collaborators with average popularity **greater than 56**, and has **more than 34** releases, they are **likely** to appear on Billboard’s Hot 100 list at least once.

### Applying learned models

**Figure 7:**
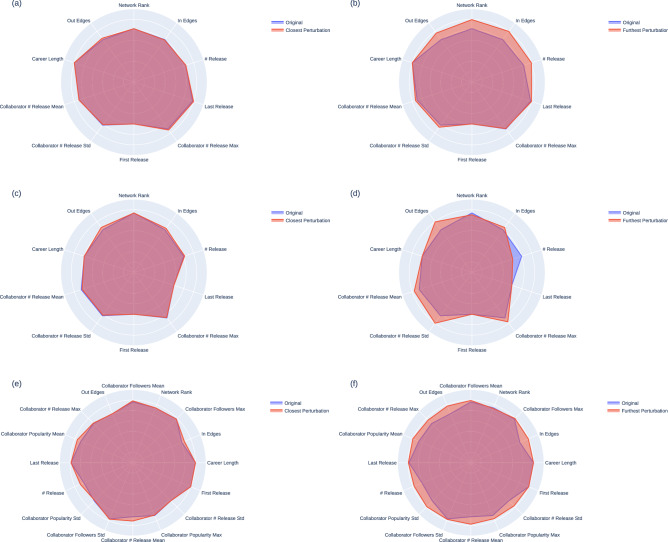
Results of applying perturbations to synthetic musicians. Figures (**a**), (**c**), (**e**) show the least amount of perturbation applied and figures (**b**), (**d**), (**f**) show the most perturbation applied that results in a prediction of (**a**), (**b**) popularity > 64, (**c**), (**d**) follower count > 504,948 and (**e**), (**f**) appearing on a Billboard’s Hot 100 list.

Once the decision trees were built for each success metric and we identified the patterns for them, we verify our observation by changing the feature values of synthetic musicians who are initially classified as ‘not successful’ to see what features affect each metric the most. To simulate real musicians while reducing our search space, we use K-Means clustering to generate 300 clusters of musicians who were labeled as **False** for each success measure. We then use the value of each cluster’s center as our synthetic data points to change. After generating the clusters, we apply 5000 random permutations to the feature values and analyze the resulting feature values using the XGBoost classifiers presented above.

For each metric, we show two examples of the same synthetic musician whose prediction changed with the least amount of perturbation applied (shortest Euclidean distance between the original and the perturbed features) and the most perturbed features (furthest Euclidean distance) out of the samples that were classified as positive.

#### Boosting Spotify popularity

Out of the 300 synthetic musicians who were predicted to have a popularity score below 64, we successfully transformed 25 of them such that they were predicted to have a popularity score greater than 64. In the examples shown in Fig. 7a and b, we find those collaboration features that boosted musicians’ popularity scores the most. By gaining more collaborators and increasing their network rank, the musicians can achieve a higher likelihood at popularity score greater than 84.

#### Boosting Spotify follower count

Out of the 300 synthetic musicians generated to have follower count $\le$ 504,948, in 51 cases, we convert them so that their predicted follower count became > 504,948. In the example shown in Fig. 7c and d, we observe that by increasing their collaboration metrics, such as incoming or outgoing edge counts, the musician is predicted to be successful, thus in agreement with the popularity result. It is notable that while the decision tree appeared to put more emphasis on the release count, the musician can increase their follower count by improving their network connections alone, even with reduced release counts.

#### Boosting Billboard appearance probability

Out of the 300 synthetic musicians, we were able to successfully change the features of 255 musicians so their prediction of appearance on the list was reversed to true. As seen in the decision tree, we find that both the musicians’ intrinsic features and collaboration features need to be improved to be placed in the Billboard list. Interestingly, we find that the quality of collaborations, such as the average follower count of the collaborators, matters more than the sheer number of collaborations, as can be seen in the example shown in Fig. 7e and f. Compared to the follower count and popularity score results, we note that many more features need to be improved for the musician to be predicted to appear in Billboard’s Hot 100 list.

**Figure 8:**
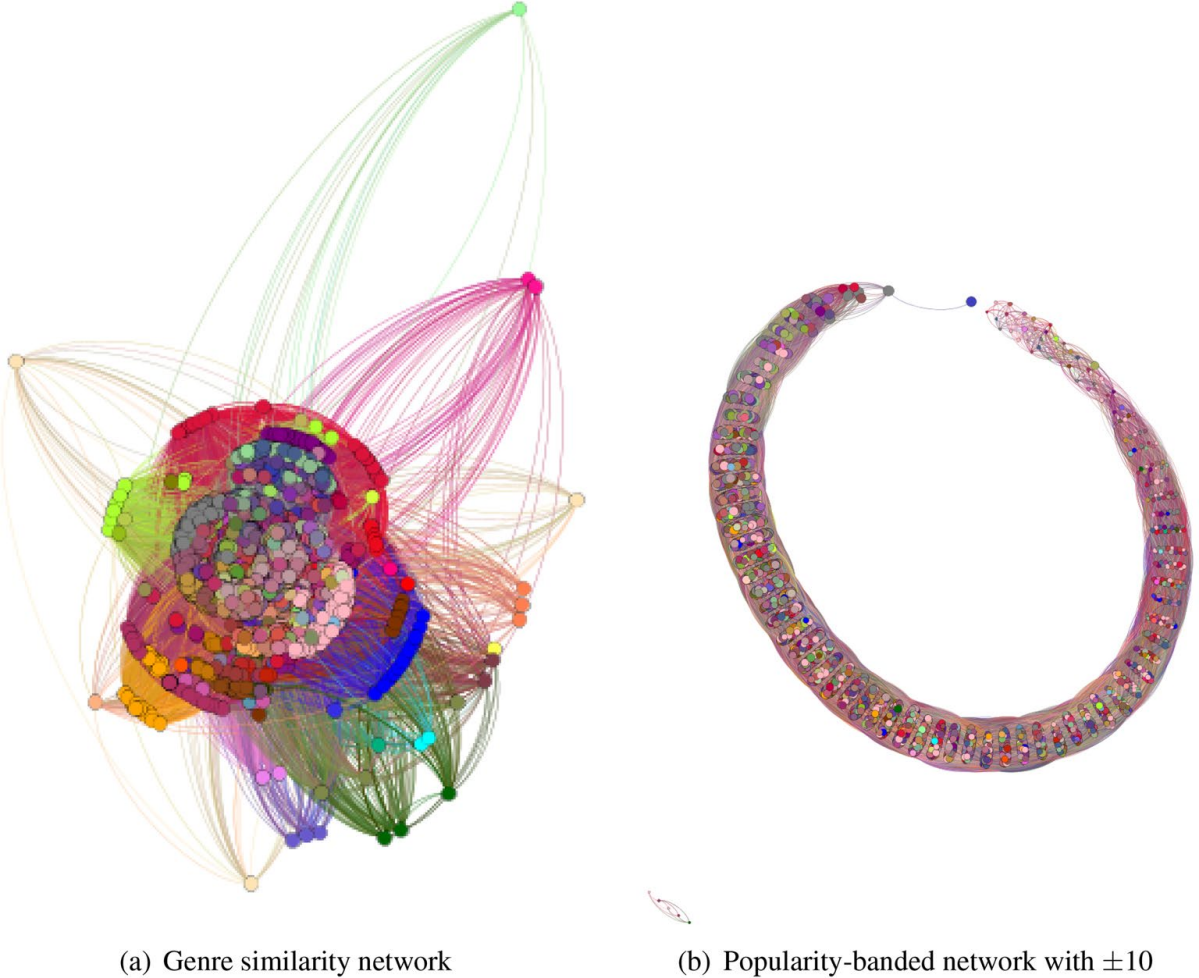
(**a**) Genre Similarity and (**b**) Popularity-banded Networks for genre-musician relation analysis.

### Genre analysis

Using the genre-based dataset, we develop two networks based on the top Spotify musicians’ genre associations. These networks provide insight into the genre-popularity relation within Spotify’s data and potential correlation between musician success and their genre association.

#### Q-model

Sinatra et al.^3^ consider the quantification of success within academia by tracking citation numbers generated by each published paper within the selected period under a specific topic classification. Within this setting, the most successful model to predict success relative to citation numbers and productivity over a researcher career uses an intrinsic *Q* parameter. This parameter stays nearly constant throughout an individual’s career. The *Q* value is defined by the equation:

$$\begin{aligned} Q_i = e^{\langle log c_{10,i} \rangle - \mu _{p}}, \end{aligned}$$

where $c_{10,i}$ denotes number of citations for researcher *i*, and $\mu _{p}$ denotes this researcher productivity value.

For our application, we extend such a definition to the field of music by using Spotify’s popularity value in place of citation numbers $c_{10,i}$ and by pulling a productivity value $\mu _{p}$ from a musician’s total release from MusicBrainz. Note that even though our *Q* parameter is more restricted due to the bounded Spotify popularity value, the *Q* value can be considered with a scaling parameter to compare with other fields or can be used by itself as a local measure relative to the music dataset.

#### Genre similarity network

The first network developed relates musicians based on the sharing of at least one genre label defined as follows. Let $G_{gs}$ be an undirected graph with no multi-edges defined by the set of musicians $N = \{a_1,a_2...a_{n}\}$ where *n* denotes number of musicians so $n=2363$ and an edge exists between musicians *u* and *v* if and only if the dot product of vectors of $G_u$ and $G_v$ is non-zero. Each node *u* is assigned two values: a weight $w_u$ ranging from 0 to 100, representing the musician’s average normalized popularity and a color representing the musician’s genre vector *G* of releases. This network is presented in Fig. 8a and shows that musician-genre relations tend to be diverse, yet they cluster around a significant group of musicians lacking in genre diversity. These clusters are connected to each other through a couple of key musicians that belong to niche genre patterns within the “other” genre subcategory.

#### Popularity-banding network

**Figure 9:**
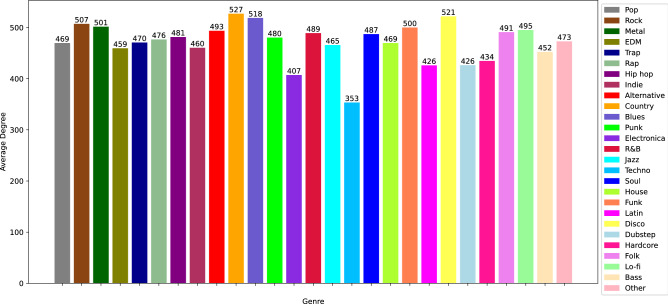
Average degree distribution for popularity-banded network.

The second network is based on the musicians’ averaged “bands” of popularity. We define this network as follows. $G_{pb}$ has the same nodes and properties as $G_{gs}$ but different edges. Let $a_u$ denote the average popularity value of node *u*. Then, an edge exists between nodes *u* and *v* if and only $a_u$ and $a_v$ are within 10 popularity points of each other. In addition, each musician is assigned the same color as in subfigure Fig. 8a,b. Figure 8b shows that the resulting network has dense layers of musicians separated into banded regions of Spotify popularity. It provides insight into the distribution of genres among Spotify’s popularity values. An interesting feature of this network is the apparent diversity of genres within each popularity band with a few lonely nodes with the maximal popularity values. Figure 9 shows average degree distribution for the nodes of this network that is consistent across all genres, with some variance appearing in some of the niches, such as “newer” genres like Techno and Electronica. The observed consistency suggests a lack of relation between popularity metrics and genre within this network. Hence, we conclude that clustering differences exist between the genre similarity network and the popularity-banded network, and there is an apparent lack of a relationship between genre and Spotify popularity values, because both networks show high connectivity and high variance in genres within bands.

## Results

Utilizing the data and network structures, we draw conclusions regarding the correlation between musicians, their collaborators, and their genre of music.

### Predicting success based on collaboration between musicians

We define a network of musicians connected by their collaboration and use the network rank in combination with other features to build a predictor model for follower count and popularity. Instead of grouping musicians to different bins as done in^5^, we use a regression approach to estimate whether the musician will meet the success criteria or not. We found that XGBoost models perform much better than other linear models, and that it can offer insights into the reasons behind a musician’s success.

**Figure 10:**
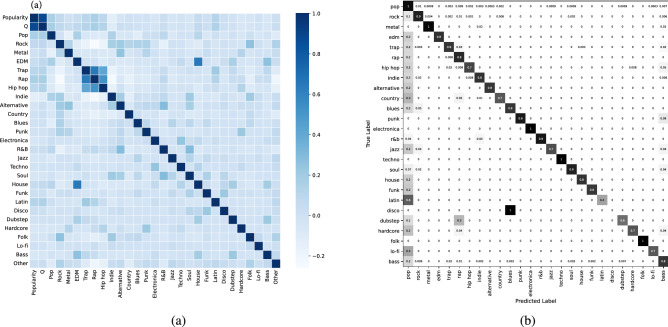
(**a**) Correlation matrix of genre-focused data, (**b**) Normalized confusion matrix for decision trees for genre prediction.

Using the popularity score and follower count as two different success measures, we find that there is a high correlation between popularity scores and collaboration history—especially so for the popularity score. However, we find that follower count is also influenced by the standalone features, such as release count or the timestamp of the first or last release.

We find an interesting observation that classical musicians have massive amounts of collaboration compared to other musicians due to their music being sampled.

We also find that the more widely accepted standard of success, appearing on a Billboard’s Hot 100 list, can be accurately predicted using the musician’s release pattern and the features of their collaborators. Our findings show that aspiring musicians do not necessarily have to collaborate with the top stars of the industry to achieve fame. In fact, we find that if the musicians themselves and their collaborators are consistently releasing music, they are more likely to reach the Billboard charts rather than those who do not.

### Machine learning model for helping musicians to improve their visibility

Among the three considered thresholds (follower count, popularity score at a certain level, and appearing on Billboard’s Hot 100 list), only the last is binary and relies on a clear criterion, being among the top 100 musicians in a certain week. We use fractions of musicians achieving such success in each of three subsets of the musician datasets (training, validation, and testing) to establish ground truth based on the same fractions of top musicians in other musician datasets, sorted by either popularity score or follower’s count.

After training the three decision trees using their training and validation data subsets, we find that by improving certain aspects of their features, a musician can raise their probability of making themselves more successful. Our examples show that as suggested by the decision trees, one should focus the most on their collaboration for popularity and overall release count for number of followers. To appear on the Billboard’s list, a musician should work on both aspects of their career, releasing more music while also collaborating with other prolific/well known musicians.

### Release normalized genres show no correlation to Spotify popularity measures under a *Q*-model

Seeking to draw conclusions relating music genres to the Spotify popularity score, we utilize the genre dataset described in the Data section by applying the *Q*-model (modified for Spotify popularity ranges) to find a correlative measure. Thus, we compute this measure using all the musicians in the genre-similarity network and their computed *Q* values from their Spotify popularity measure along with their binary vector *G*. Figure 10a shows the results that reveal a high correlation between popularity measure and the *Q* value in our modified *Q*-model. We observe strong correlations between specific genres such as Trap, Rap and Hip Hop, arising due to the commonality of pairing of these genres in collaborations of several musicians. Unexpectedly, we observe close to no correlation between any of the genres, the Spotify popularity measure and the *Q* parameter. This suggests that the popularity distribution within our genre dataset is even across all genres. This is confirmed from the *Q*-model perspective by lack of any indication in the Spotify data that a specific genre will increase the likelihood of success in music in terms of Spotify’s popularity measure.

Finally, we examine the popularity distribution across our genre dataset. In Fig. S4 in the Supplementary Materials presents the results that show the mean and variability for each song and their respective genre vector. Thus, we see a consistent midpoint around fifty and sixty popularity points for each genre, which is above the average since there are only top 100 musicians in the dataset. These results are consistent with our correlation analysis, showing that the average Spotify popularity scores vary slightly across the genres. An interesting data point shows the maximal popularity score among the Pop and Soul genres, at the time the top song on Spotify is of these genres. This observation prompts the interesting question whether a classification of Top 100 songs is strict enough to see such correlation between genre and popularity. Thus, further analysis into maximal popularity songs and their respective musicians could identify favored genres within a single popularity band of “hit songs”.

### Predicting genre based on Spotify measures

To further observe potential for correlation between musician average popularity values and genres, we employ machine learning methods to predict genres from the Spotify measures. We employ three types of classifiers for such predictions: decision trees, k-nearest neighbors (k-NN), and support vector machines (SVM). For each model, we use the genre dataset described in the Data section consisting of each musician, their average popularity score from their top ten songs, their associated *Q* value and their productivity. We define productivity as the number of all-time releases by an artist throughout their career. For training, we include musicians’ binary vectors *G*.

Accuracy is measured for each model against the partition of our genre dataset, yielding scores of about 25% with SVMs, 60% with k-NN and 82% with decision trees. The high accuracy in correct genre prediction using decision trees implies a complex, but strong relationship between popularity, *Q* values and genres. Figure 10b displays the normalized confusion matrix for our multi-class decision tree. The high number of false predictions for the pop genre is caused by the broad distribution of the pop genre across our dataset and the generally high levels of success of pop music across multiple audiences. We also consider joining multiple niche genres such as bass, blues, jazz, and soul into one complex genre. Intuitively, we need complex genres because musicians often release music with different genres. The conclusion is that while genre does not show any direct correlation to success measures relative to Spotify data, there does exist predictability in the complex relationship between these success measures and specific genres under a decision tree model.

## Limitations and future works

This work focused on analyzing the success of professional musicians and ways for less-successful musicians to improve their career profile to become such. While we were able to find some interesting results from our work, there are extensions of this work that can address its limitations in the future. With more historical data, it will be interesting to find out how long the classifiers stay valid before they start failing. We find that career length plays an important role in the success of musicians, especially for follower count and appearing on Billboard’s Hot 100, as shown in our correlation analysis and the resulting classifiers. Although our dataset contains the timestamps of each release of every musician, we were not able to find a stable data source for accounting for a stream count/record sales of all these releases. Thus, we were not able to gather much data on the specific success of each release present in the dataset. Spotify also does not provide the historical values of popularity score or the follower count, making it impossible to understand the impact of each release on a musician’s metric. Future work could extend upon this work by collecting data over a longer timeframe (e.g., a year) to collect the changes to the musicians’ success metrics and their release frequencies. Another interesting future work would be to divide the musicians into separate groups, e.g., by genre, and apply similar training/testing procedures for each group.

## Discussion

In this work, we seek to explore various ways we can represent the music business in a network structure, as well as the correlation between success metrics with the network features in combination with musician features collected from the Musicbrainz database and the Spotify API. We find that success, which is represented by Spotify’s popularity score and number of followers, can be predicted using simple classifiers that are human-readable. Thanks to the time-agnostic nature of these models, the same trained classifiers could be re-used to predict the success of musicians in coming years. We also find that there is not a strictly linear correlation between the musicians’ genres and their success. However, using tree-based classifiers, we observe that there exists a complex relationship between a musician’s success and the genres to which this musician releases music. We find that classical musicians still permeate the music business hundreds of years after their time of release, and that success measures of musicians can be predicted using publicly available data. We also find that while collaboration between musicians may improve one’s popularity score on Spotify, it impacts the musician’s overall follower count only indirectly by increasing their popularity score. We also find that musicians who collaborate with less known musicians can also get a place on the Billboard, given their collaborators are prolific enough.

Our approach can be generalized and applied to any type of network data that has a target metric. It can also be extended to create a recommendation system for aspiring musicians, providing them with hints on how to raise their visibility on a given platform, such as recommended collaborations or the number of releases. Future work can extend this by adding more data to the network or considering different classifier models. Spotify does not currently provide an ‘all-time’ measure for popularity or the number of streams each song has. Having access to this set of more detailed data may enable researchers to gain more interesting insight about what makes a musician more successful than their peers.

## Supplementary Information

Supplementary Information 1.

## Data Availability

The data is available by sending a request to I.K. E-mail: inwon.kang04@gmail.com The data and research that produce it was reviewed and classified as exempt by The Rensselaer Polytechnic Institute (RPI) Integrated Institutional Review Board as the research involving the collection of existing data from sources that are publicly available.
