# Supplementary material for: Analyzing and predicting success of professional musicians
Source: Sci Rep. 2022 Dec 17;12:21838. doi: 10.1038/s41598-022-25430-9 (PMC9759548; doi:10.1038/s41598-022-25430-9)
Supplement: Supplementary file 1 — Supplementary Information 1. [file 41598_2022_25430_MOESM1_ESM.pdf]

Supplementary Materials for
Analyzing and Predicting Success of Professional Musicians

Inwon Kang, Michael Mandulak, Boleslaw K. Szymanski

Figures S1-S3 and Table S1

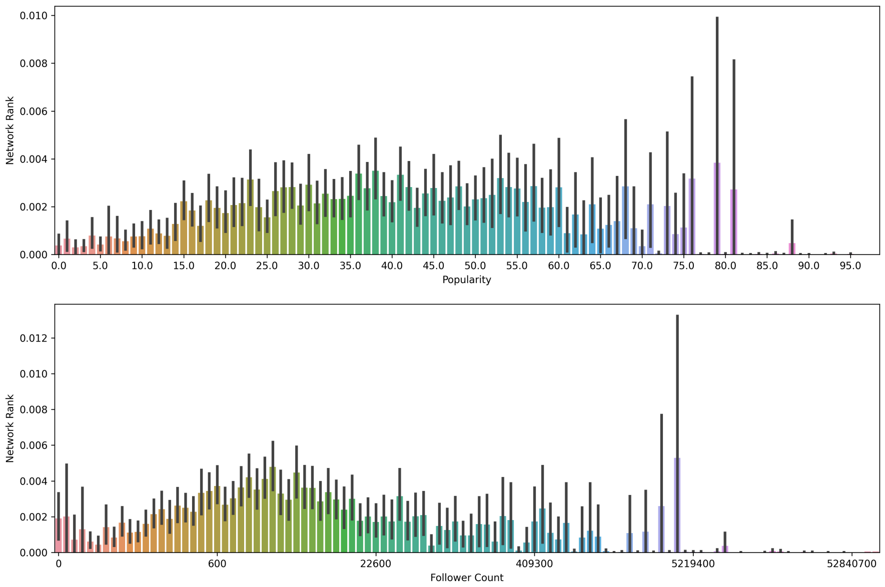

Figure S1. Popularity Score and the Number of Follower correlation with professional musician's Network Rank

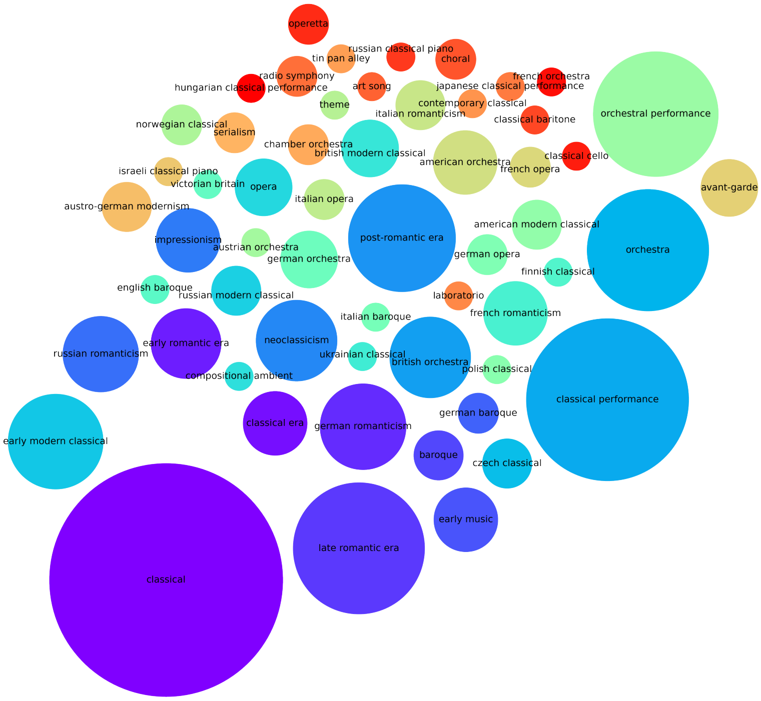

Figure S2. Top 100 genre tags according to the network rank of professional musicians in Collaboration Network

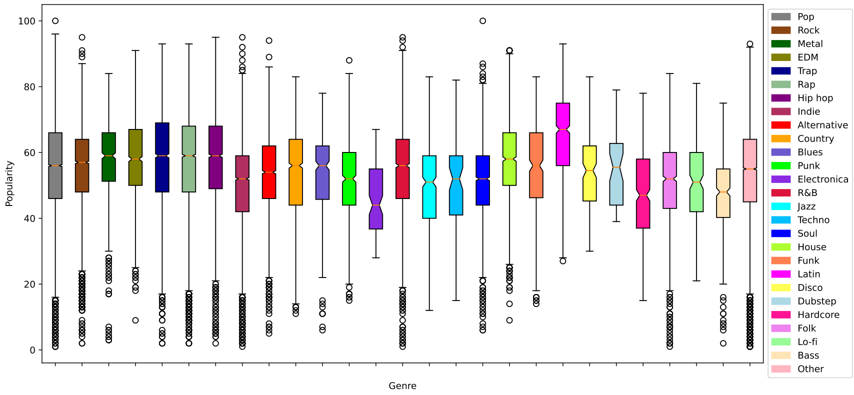

Figure S3. Popularity distributions within genre-parsed data

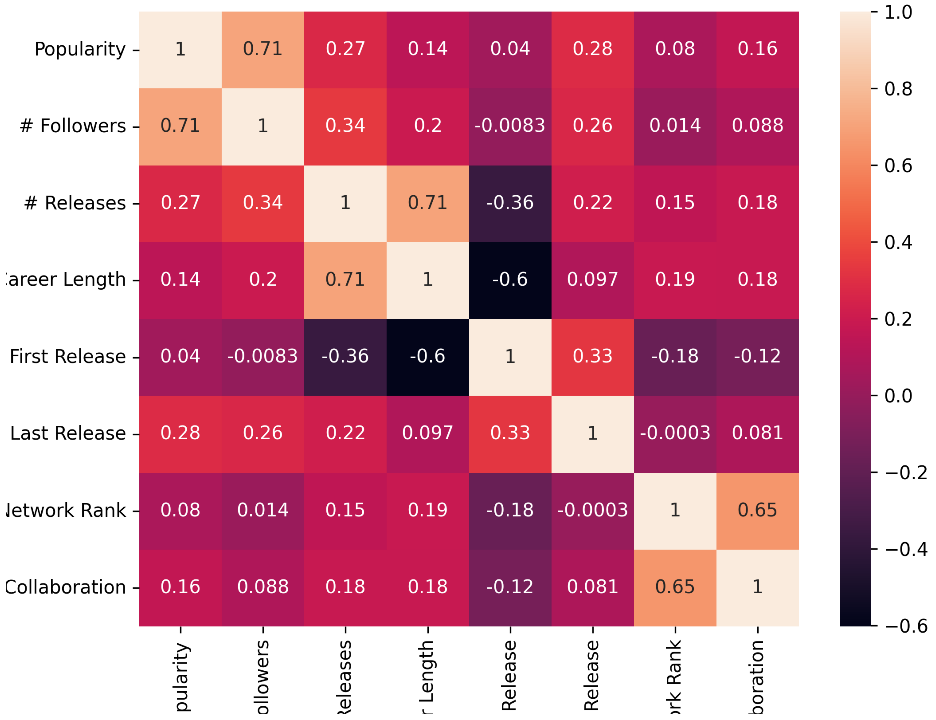

Figure S4. Kendall Tau's correlation between features used in models

Features	Popularity Score	Number of Followers	Billboard's Hot 100 appearance
Popularity Score			✓
Follower Count			✓
Network Rank	✓	✓	✓
Collaborations Received	✓	✓	✓
Collaborations Given Out	✓	✓	✓
Release Count	✓	✓	✓
Career Start	✓	✓	✓
Career End	✓	✓	✓
Career Length	✓	✓	✓
Maximum of Collaborators' Release Count	✓	✓	✓
Average of Collaborators' Release Count	✓	✓	✓
Standard Deviation of Collaborators' Release Count	✓	✓	✓
Maximum of Collaborators' Popularity Score			✓
Average of Collaborators' Popularity Score			✓
Standard Deviation of Collaborators' Popularity Score			✓
Maximum of Collaborators' Follower Count			✓
Average of Collaborators' Follower Count			✓
Standard Deviation of Collaborators' Follower Count			✓

Table S1. Features collected from the dataset used by each classifier

**Max Depth**	$[3,4,5]$
**N Estimators**	$[1]$
**Max Leaves**	$[4,5,6,7,8]$
**Alpha**	$[0,10,20,30,40,50]$
**Lambda**	$[1,10,50,100,1000]$

Table S2. Range of tested hyperparameters for XGBoost

Metric	Random State	Max Depth	N Estimators	Max Leaves	Alpha	Lambda	learning_rate
Popularity	0	4	1	8	0	0	0.1
Follower Count	0	4	1	7	0	0	0.1
Billboard's Hot 100	0	4	1	6	0	100	0.9

Table S3. Hyperparameters used to train the final XGBoost tree for each metric
